# Nursing students’ perception: Escape Room use in teaching leadership skills

**DOI:** 10.1590/0034-7167-2023-0414

**Published:** 2024-07-19

**Authors:** Chennyfer Dobbins Abi Rached, Emili Amani Alves da Cruz, Maria Helena Cardoso da Mota, Caroline da Silva Fonseca Paulo, Giovanna Micucci Pires Amaral, Daniela Lika Nakajima

**Affiliations:** I Universidade de São Paulo. São Paulo, São Paulo, Brazil

**Keywords:** Leadership, Teaching, Gamification, Nursing, Perception, Liderazgo, Enseñanza, Gamificación, Enfermería, Percepción

## Abstract

**Objectives:**

to understand nursing students’ perception regarding Escape Room use to develop leadership skills.

**Methods:**

a qualitative exploratory-descriptive study, with 97 nursing students. Escape Room game sessions were held, totaling ten. Subsequently, a debriefing and analysis was carried out using the Discourse of the Collective Subject method, through the focus group technique. Ethical procedures were respected.

**Results:**

students’ perceptions about using this methodology to develop leadership skills were positive and significant. The effectiveness of the game in teaching and developing these skills in nursing practice stands out.

**Final Considerations:**

the game was an effective active methodology in approaching the proposed content, promoting satisfaction and easy assimilation.

## RESUMEN

**Objetivos:**

comprender la percepción de estudiantes de enfermería sobre el uso del *Escape Room* para desarrollar habilidades de liderazgo.

**Métodos:**

estudio cualitativo exploratorio-descriptivo, con 97 estudiantes de enfermería. Se realizaron sesiones de juegos de *Escape Room*, en total diez. Posteriormente, se realizó un *debriefing* y análisis mediante el método del Discurso del Sujeto Colectivo, mediante la técnica del grupo focal. Se respetaron los procedimientos éticos.

**Resultados:**

las percepciones de los estudiantes sobre el uso de esta metodología para desarrollar habilidades de liderazgo fueron positivas y significativas. Se destaca la efectividad del juego en la enseñanza y el desarrollo de estas habilidades en la práctica de enfermería.

**Consideraciones Finales:**

el juego resultó una metodología activa eficaz en el abordaje de los contenidos propuestos, promoviendo satisfacción y fácil asimilación.

## INTRODUCTION

Nowadays, the healthcare system has been evolving towards a new format based on primary care, focused on home and community care, in addition to hospital services. This change strengthens the role of nurses as leaders of nursing staff^(1)^. However, few nursing professionals argue that leadership is required to develop better quality of service and efficient care provision. The literature on nursing education suggests a need to develop professionals’ awareness of the need for leadership as well as expanding pedagogical methods for developing leadership skills in nurses^(2)^.

In 2018, changes occurred in the Brazilian National Curricular Guidelines (DCN - *Diretrizes Curriculares Nacionais*) for nursing courses, directing professional training towards compliance with the Brazilian Health System (SUS - *Sistema Único de Saúde*) principles and guidelines. For this to occur, the teaching-learning process needs a change, with a focus on encouraging creative potential, autonomy and self-management of learning. Therefore, it is recommended to use an innovative approach that provides integration skills into the reality of health work. Therefore, it is necessary to use diverse technologies in the process of building knowledge^(3)^.

The Escape Room or breakout is considered a serious game based on the insertion of players into a physical or digital playful space, with the purpose of solving a problem through clues. As it is versatile, it has been applied to teaching to acquire knowledge and skills in different areas of knowledge^(4)^. In Brazil, such terms are included in this emerging methodology called gamification, which aims to implement game elements in activities other than games, such as in the classroom^(5)^. Thus, the Escape Room emerges as a teaching tool in the teaching-learning process, with documented experiences in teaching different areas, such as health, computer science^(6)^, mathematics and languages^(7)^, among others, with similar results, in which participants were more motivated and interested in activities adapted to certain settings.

Entering nurses’ professional reality, the Escape Room strategy was recently chosen to carry out an awareness-raising and educational activity on pressure injury prevention in a philanthropic hospital in São Paulo, which had the participation of several healthcare professionals, such as nurses, nursing technicians, nutritionists and physiotherapists^(8)^. Analyzing the results and discussions produced by the research, it was possible to see how even in professional practice this strategy is highly effective and satisfactorily meets the objectives it sets out to achieve.

Furthermore, studies also demonstrate the wide usability of an Escape Room, using it for other strategies. At an engineering university in Italy, it was observed that, due to the university dynamics, students did not socialize much with each other, meaning they did not develop crucial aspects for their personal and professional lives. Thus, the active methodology was applied with the aim of promoting socialization between them, teamwork, communication skills, among others. Even though it comes from different contexts, this educational environment was also assessed as very satisfactory by participants^(9)^.

Studies show that there is greater interest in wanting to learn more about a certain subject when using a playful method^(4,9-10)^. In this aspect, it is believed that the game can be a strategy for learning leadership skills during nursing training, since, according to the Brazilian National Curricular Guidelines, leadership is one of the essential skills for healthcare professional training that must be built throughout their higher education^(11)^. In this regard, since students are actively finding ways to solve a common problem within an Escape Room, they are also developing essential skills and competencies for their professional performance.

Therefore, this study aims to answer the following research question: what is the nursing students’ perception regarding Escape Room use to develop leadership skills?

## OBJECTIVES

To understand nursing students’ perception regarding Escape Room use to develop leadership skills.

## METHODS

### Ethical aspects

All ethical procedures were respected, for Brazil, based on Resolution 466/2012. The project was approved by the *Universidade de São Paulo* Nursing School (EEUSP) Research Ethics Committee. It was presented to participants, explaining the importance of collaboration for deepening and understanding the topic and clarifying the freedom and willingness to participate without any type of prejudice or sanction. All participants signed the Informed Consent Form (ICF).

### Theoretical-methodological framework

Discourse of the Collective Subject (DCS) analysis was used^(12)^.

### Study design

This qualitative study, with an exploratory-descriptive character^(13)^, made it possible to deepen knowledge about the topic studied and expose research subjects’ perceptions and opinions about the reality they experienced. To prepare the manuscript, the COnsolidated criteria for REporting Qualitative research (COREQ)^(13)^ recommendations were followed, an instrument made available by EQUATOR network.

### Methodological procedures

Data were collected in a debriefing session, using the focus group method, which involves a research approach^(14)^ that employs stimulus resources, often interactive activities, to promote and maintain group conversations. These conversations facilitate exchange of knowledge and experiences among participants. In this regard, the Escape Room was played and, at the end, a debriefing session was held to understand students’ perception of aspects involving teamwork and leadership developed throughout the game. All activities were carried out according to schedule.

### Study setting

The setting was initially two meeting rooms, both measuring around 20 m^2^, with capacity for up to ten people, with access doors for the entry and exit of participants and a door between the two rooms, with a table, chairs and some cabinets. The windows of both rooms gave access to a corridor, which is why they were covered to maintain the privacy of the game, the participants and the researchers. Then, due to the greater game ease, agility and experience, simulation began to take place in a simulation room in a 20 m^2^ house with capacity for up to ten people. The room had: access doors for participants to enter and exit; three distinct rooms (living room, bathroom and bedroom) with access doors to each one; living room containing sofa, dining table, sink, refrigerator, washing machine, sink and cabinets; bathroom containing shower, toilet and sink; bedroom containing bed, desk and a closet; windows that looked out onto the outside of the room. There was a control and observation room integrated into the simulation room, in which, using two notebooks, images and sounds were broadcasted via Google Meet^®^, present in both settings, equally. Therefore, it was necessary to prepare the rooms so that they could receive the game. In this way, the researchers met about 30 minutes beforehand to prepare the room. The windows were covered with kraft paper so that there was no interference from the external environment. The study took place at the nursing school of a public Higher Education Institution, located in the center of the city of São Paulo, SP, Brazil.

### Data source

The study was developed with 97 students, intentionally selected, who were undergoing their nursing degree. The number of participants was distributed between seven and ten participants in each game, totaling ten games. Soon after finishing the game, this same group of participants was directed to a focus group, in which perceptions about the Escape Room were discussed. The total number of participants was delimited by the characterization of repetition of speeches, making it possible to achieve an understanding of the phenomenon studied. Participants were identified by sequential speech number in the focus group (*sub_1, sub_2*). Inclusion criteria included undergraduate nursing students, nursing school students between the first and last semester of graduation so that there was no homogeneity in the focus groups, as students from different semesters were randomly placed in groups. Even with the sample non-homogeneity, the same challenges and game assessment criteria were applied to all students who participated in the research. The only variable between the groups was the number of additional clues that each group received, according to the time they were taking to complete the Escape Room. Exclusion criteria covered anyone who was not in the nursing course at the nursing school.

Participants were recruited through invitations via digital (WhatsApp^*®*^ and email) and interpersonal means in classrooms. To participate in the game, it was necessary for a person to express interest so that preceptors could organize the game calendar according to participants’ demand and availability. If there was a withdrawal on the day of the game, they were relocated to another day when the game would be played again. To select our source of data, we chose to capture it through a debriefing, applying the focus group technique and a questionnaire because, at the same time as the first one explored the subjectivity of the results of the activity and encouraged them to reflect, the other served as an aid to analyze this subjectivity more objectively, capturing topics that we sought to discuss and to allow us to have a better statistical view.

### Game presentation

The sessions were organized so that there was enough time to carry out both the Escape Room and the debriefing moment to apply the focus group technique. All preceptors played Escape Room in a specific space, understanding the dynamics and form of assessment.

Prior contact was established before the start of the game with participants for a brief explanation of the game, its duration and how preceptors monitored it, to sign the ICF and communicate that at the end of the game a debriefing session would be held. The game had a maximum time of 60 minutes to complete, consisting of two stages, each with its own story and clues, which were distributed in the room. As the game took place, the researchers were always attentive to the difficulties and abilities that the groups presented, in such a way that, for some groups, more clues were given (dropped under the door) to complete the game. The game’s challenges and clues were related to the fictional story of a laboratory professor who allowed a contagious virus to spread in his laboratory, which is now locked with students inside, and they need to discover the clues left by the professor to leave this room and save themselves from contamination. At the end of the game, all participants in the same game met with the researchers so that debriefing could be carried out.

### Data collection and organization

In this research, ten group sessions were held, with a schedule agreed among participants from December 2022 to March 2023, held shortly after the Escape Room. No sociodemographic data were collected. Only the researchers carried out data collection, with prior training and discussion among them to define the best methods for this purpose. Participants’ verbal responses were not recorded, only written down in more objectively, considering that a written questionnaire was also used and was filled out by all participants in the focus group. The focus group was planned according to the following steps: composition (participants, coordination staff, recruitment); tools (setting and topic guide); operationalization (structuring); and assessment.

### Work stages

#### Composition

Participants: 97 undergraduate nursing students who participated in a complete session of the Escape Room. Some of the participants had a previous relationship with the staff, however this did not influence the implementation of the focus group;

Coordination staff: composed of a nursing professor who focuses on leadership research, plus four nursing students who participated in prior training for data collection. The staff was responsible for directing the discussion about the game with participants and recording such perceptions;

Recruitment: an invitation to the focus group was given before the start of the Escape Room. There were no free choice criteria for participation in the focus group, nor the right to refuse or withdraw. The objectives, methods, risks and benefits of the focus group as well as the topic that would be discussed were explained to participants.

#### Tools

Setting: the focus group took place in the same room as the first part of the game in the first setting. Afterwards, when there was a change of setting, the focus group took place in the control room. Participants and moderators were arranged in the focus group at a round table, all seated. Participants were welcomed at the end of the game, and the researchers present welcomed participants into the room to carry out the focus group;

Topic guide: the topics in the guide were created by the authors based on the analysis of the main topics covered regarding “leadership”. Also, the main factors that were necessary for a good conclusion of the dynamic were analyzed after a test session with moderators and authors. The guiding questions were: how do you perceive teamwork (did they go out alone or individually, what was the collaborative work like)? Were there conflicts? How were they resolved? Was there a leader on the staff and what was that leadership like? What is the perception regarding staff members? Did they have an active role in solving problems that would be achieving the game’s goal, which was escape?

#### Operationalization

Structuring: there were ten meetings for the focus group, with the same participants who made up the group for the Escape Room. Each meeting lasted around 30 minutes. As the focus group took place right after the games, there was no “icebreaker” dynamic among participants. The researchers were responsible for moderating the group, asking students to remember and detail the game content, bringing insights and applied skills. After participants ran out of memories, aspects and concepts of collaborative leadership were discussed. Participants’ statements were transcribed by the researchers at the same time as the focus group. In the room where the focus group took place, there was only the presence of participants and the researchers responsible for moderation.

#### Assessment

The assessment guide applied at the end of each session was used in order to identify how the focus group was conducted from participants’ perspective. This instrument was composed of questions related to place and time, operationalization (objectives, game topic, conducting participatory discussion and synthesis), role of moderator and observers. The answer possibilities were on a Likert-type scale, being: 0 - Not applicable at all; 1 - Applied to some degree or for a short time; 2 - Applied to a considerable degree or for a good part of the time; 3 - Applied a lot or most of the time. A class and training were given to apply the instrument. The analysis of results showed adequacy of the method.

#### Instrument construction

The instrument was developed by the authors themselves, and the instrument content was subjected to validation by leadership experts (judges). Construction and validation were based on three poles (theoretical, empirical (experimental) and analytical (statistical)). The theoretical pole substantiated the properties and dimensions of the objects of study. Assessment was carried out according to three criteria (clarity, relevance and appearance). The judges assigned values from 1 (not at all relevant) to 5 (very relevant) for each of the items. Value 1 was the worst score, and value 5, the value the best, whereas those with values greater than or equal to 4 indicate that the item corresponded to the proposed objective. To analyze the instrument content, the Content Validity Index (CVI) was used, which measures the proportion or percentage of judges who are in agreement on certain aspects of the instrument and its items. The index score was calculated by summing the agreement of items that were marked as “4” or “5” by experts. The level of agreement considered acceptable was a CVI for each item greater than or equal to 80%. The CVI^(15)^ was used to determine the validity of the questionnaire items. CVI scores ranged from 0.74 to 1.00, and the total CVI was 0.93, indicating adequate content validity. Pre-test carried out in the Escape Room, consisted of 12 participants.

#### Data analysis

Data were collected and transcribed immediately after the Escape Room sessions, during a round table with the volunteers. One of the moderators who followed the game was responsible for transcription, which was later shared with the authors. Topic identification and selection occurred based on the analysis of volunteers1 statements. This information was analyzed and interpreted following the DCS^(12)^ method, which allowed the reconstruction, through clippings of different individual speeches, a kind of puzzle, representing the synthesis speeches considered essential to express participants’ collective thinking in this study about the phenomenon investigated.

## RESULTS

According to statements during the focus groups, students realized that the Escape Room enables the application of leadership skills, such as teamwork, communication, decision-making, planning, creativity and persistence. It was observed that this method can be used as an educational intervention in teaching nursing leadership skills, since reflections brought up aspects that favor the exercise of leadership by nursing staff, such as engagement, teamwork, time and conflict management. In the interpretation phase, data regarding leadership skills that are applied during the Escape Room, from the perspective of students who participated in the game, were grouped by categories of Central Ideas (CIs). They will be presented below in the form of DCS.

### Teamwork

In this CI, interviewees demonstrated that teamwork is essential to achieving the game’s objective. Most participants were able to value relationships with others, collaborative work, and understand aspects of problem management, the impact on our actions, as well as how it can affect the scope of work, thus trigegring reflections regarding changes in behavior both in the way of leading and in the way of operationalizing work processes.

*When we helped each other and thought as a group, figuring out the clue was faster.* (sub_11)

*Thinking in groups is much easier and quicker than we would think alone.* (sub_84)

*When everyone started working together, that’s when things started to flow.* (sub_90)

### Leadership

In the topic of leadership, a significant part of participants reported, initially, that they did not notice the prominence of an individual as a leader, but rather that at various times different people stood out, guiding the group to solve the proposed puzzles. Many also reported that they missed the approach to leadership during their undergraduate studies and that they enjoyed having practical contact with the topic.

*I thought it was really good to bring a new methodology to teaching nursing. Leadership is a vague topic for us students, and thinking that a leader needs to achieve goals and inspire followers is something that makes perfect sense at Scape.* (sub_41)

*At the time of the game, I ended up not noticing much because of the excitement, but now I think having a leader was essential for teamwork to work.* (sub_12)

*When you begin to understand the role of nurses during undergraduate degree, leadership even appears as a necessary skill, but it is very difficult to learn this in theory. With the Escape, it seems like it made more sense.* (sub_19)

### Feelings towards the strategy

Students expressed the positive and aggregative meaning of the gamification strategy for university education in terms of leadership competency.

*It seems that, when everyone was engaged in this activity, several strategies began to appear for us to solve the problem, it was very interesting.* (sub_25)

*It goes beyond the leader, I think. I think the strategy came about with everyone working together thinking of a way to reach a resolution.* (sub_76)

### Communication

In all sessions, there were participants who demonstrated good communication, expressing satisfaction with how the group talked when faced with an obstacle.

*Leadership needs communication and interpersonal relationships. I felt that it is fundamental to achieving the game’s objective, I think it is related to leadership practice.* (sub_87)

*At the beginning of the game, everyone was a little quiet, each one doing something, but we realized that we would only be able to unravel the clues if we communicated, so, for me, I think that the person who took the initiative to start effective communication with everyone can become a good leader.* (sub_56)

### Decision-making

The results showed that participants realized the importance of a leader in the decision-making process under pressure, highlighting the need for quick decisions to achieve objectives. Furthermore, it was observed that the decisions made were collaborative, with all group members contributing suggestions and opinions.

*The decisions were all made together. Someone would make a suggestion and everyone would give their opinion.* (sub_15)

*When everyone was helping each other, making decisions became easier, as the leader of our group expressed interest in each person’s opinion to take the next step.* (sub_66)

### Planning

During the experience, nursing students reported two different perspectives regarding planning. Some participants mentioned that the process was chaotic, indicating that there was no effective planning during the game. On the other hand, other students noted that one person took on the role of leader and took charge of planning, which resulted in a more organized and focused experience.

*Wow, we had no planning, chaos.* (sub_22)

*I felt that someone took over planning and held our attention, favoring the planning of where we would go.* (sub_66)

### Creativity

Students emphasized the importance of creativity to uncover clues and overcome challenges in the game. Group collaboration was also highlighted as essential for boosting creativity, as each member brought unique ideas, enriching problem solving.

*We needed to be very creative to uncover the clues, thinking as a group also helped, because everyone had an idea.* (sub_74)

*Everyone helped everyone, it was a brainstorm at one point.* (sub_69)

### Persistence

Participants reported situations that demonstrated their persistence in the face of the challenges presented. One of the participants expressed having faced moments of confusion and lack of motivation, even considering giving up. However, the moment the staff managed to uncover a clue, this feeling of victory provided “a magical and rewarding moment”. Another student mentioned that he also faced significant difficulties, to the point where he felt like giving up and lying in bed. However, his teammate played an important role in encouraging and motivating him to continue. The feeling of achievement and victory at the end of the game highlighted the importance of cooperation and mutual support among staff members.

*I tripped in the game, I didn’t understand anything, so I wanted to give up, but when the encoder alarm went off and we got it right, it was a magical moment of victory.* (sub_24)

*There was a time when I wanted to lie down in bed, man, very difficult, but my colleague pulled me and said “let’s go”, I think it has to do with the role of leader.* (sub_69)

## DISCUSSION

Upon applying Escape Room, it is primarily clear how teamwork is a strong point of the game and perceived by players. In the activity, together, they discover clues, solve puzzles and perform tasks in one or more rooms in order to achieve a specific objective (which is usually escaping the room) in a set amount of time^(16)^. Its usefulness becomes evident when comparing this active Escape Room methodology with the more traditional ones that involve human resources in groups. Participants were actively involved in the activities, and the energy level was significantly higher^(17)^.

Even more interesting is to associate teamwork with the profession, in which many nurses today are faced with conflicts with the staff, lack of knowledge about who their co-workers are and their capabilities, insecurity in getting along, all of this is often associated as a product of the problems mentioned above, as an overloaded and often extremely rotating work environment brings many difficulties to group dynamics. Hence, the Escape Room showed that it has the capacity to make people better understand each other, each other’s capabilities and come together for the same objective, demonstrating broad usability for nursing undergraduate students^(18)^.

Furthermore, more than being able to work in a staff, nurses are often in the position of leading it. The concept of leadership occurs through the process of interactions, in which leaders are responsible for influencing those who follow them, in order to carry out actions with the aim of achieving objectives in their own contexts^(19)^. In the applied game, it was possible to observe that many participants experienced their self-knowledge regarding leadership and also their ability to identify it in other people.

Therefore, developing their leadership capabilities since graduation is a fundamental pillar for many scenarios to be avoidable. The Escape Room includes all students as potential leaders, and this is important, thinking about a manager and their staff, as it makes relationships more horizontal. A good leader provides opportunities for their group and recognizes its efforts^(20)^, forming opinions in staff, managing people, and the behavior and power practiced influence different aspects of the work environment and relationships. Developing the leadership of undergraduate students helps in their performance as professionals. Leadership is covered during nursing courses in some disciplines superficially; therefore, providing this opportunity to students was a strategy for training professionals who are more prepared and aware of the creation of positive workplaces that promote workers’ well-being and engagement. This game put everyone in active positions to seek knowledge and critical reasoning, in an attempt to make people feel recognized and confident, making their engagement better^(20)^.

Consequently, there are several skills that Escape Room seeks to improve in players, and one of them is creativity, which favors effective communication, expanded vision for problem-solving, encouraging critical reasoning. In the game sessions, it was possible to analyze, in students’ perceptions, the presence of these skills and their satisfaction with the educational strategy in this regard, demonstrating its effectiveness.

Creativity is one of the essential leadership skills in nursing. Creative nurses identify and seek innovative solutions, improving healthcare and motivating both nursing and healthcare staff to provide safe, quality care. Nursing leaders can use creativity to improve operational/care processes, developing efficient protocols, introducing new technologies and implementing strategies to reduce iatrogenic injuries^(21-24)^. Therefore, effective communication is essential in the healthcare context, as it helps ensure that information is transmitted in a clear and understandable manner to the staff^(25)^.

Nurses make decisions both in relation to care management and in relation to staff management, solving complex problems and seeking to create a work environment that promotes patient safety and staff well-being. In this aspect, creativity and leadership are intertwined in the search to solve the challenges of everyday nursing, such as resource allocation, optimization of patient care processes, conflict management, among others that make creativity essential to identify innovative solutions to problems^(24-27)^.

Furthermore, the decision process has great relevance for effectiveness and success within institutions, as it is essential among a leader’s skills, being a process with problem-solving potential, allowing reasoning about which path to take and which choices will bring greater benefit to the staff and organization^(28-29)^. In Escape Room, students are often placed in positions where decision-making is necessary to solve the game, so there is an important booster in this regard, noticed by many of them in our experience.

Critical thinking about care, with theoretical and practical foundations, is the responsibility of a nursing staff, to be trained with innovation and creativity about better methods, means and, mainly, and prepared for decision-making for this, which directly implies quality of care in the care to whom the services are provided. All of this is inherently linked to the planning capacity, a function exclusive to nurses, in accordance with the socio-technical divisions of work and which, as a skill, is a fundamental pillar so that processes within the health service can occur successfully and in an organized manner^(30)^.

Thus, it is noted how this fact brings to light how many perspectives and faces of situations are very related to the ability to carry out planning in nursing, in addition to how this affects third-party situations as well, such as error prevention, the correct and efficient use of resources, the satisfaction of those receiving care^(31)^. It is interesting that many students did not realize, in advance, all their game strategy planning, in which several of the previously mentioned skills were used, so that, from debriefing, they could reflect and analyze how their capabilities connect in an important way to carry out effective work.

Finally, Escape Room use proved to be a fundamental element for students’ personal and collective growth. The educational Escape Room follows the principles of gamification, i.e., it is based on functioning, aesthetics and playful thinking to entertain people, encourage activities, provide learning and problem-solving with dynamics and mechanisms particular to games^(7)^.

In short, gamification shows to have important potential for increasing students’ motivation, contributing to a better understanding and resolution of problems^(32)^ that they will encounter on their paths. This occurs by using the skills that will be able to develop with the strategy and, thus, contributing to the training of more qualified professionals and more effective healthcare systems.

### Study limitations

The study was limited by the origin of students used so that they all came from the same institution and only from the nursing course, which may have an influence on the results related to the gamification methodology.

### Contributions to nursing

The research provided evidence of the potential of teaching methodologies with behavioral modeling for developing collaborative leadership in nursing, identifying possibilities for the applicability of this methodology in undergraduate course subjects, not just nursing, but other courses and other topics, such as interprofessional work, organizational culture, among others, strengthening quality training.

## FINAL CONSIDERATIONS

Through the Escape Room, leadership learning was facilitated. Nursing students perceived leadership as an essential managerial skill for developing teamwork, solving complex problems and conflicts. The greatest learning difficulties were related to persistence and planning skills, however, overall, students demonstrated satisfaction with the proposed methodology and an easy assimilation of suggested contents.
