# Supplementary material for: Nursing students’ perception: Escape Room use in teaching leadership skills
Source: Rev Bras Enferm. 2024 Jul 19;77(2):e20230414. doi: 10.1590/0034-7167-2023-0414 (PMC11259438; doi:10.1590/0034-7167-2023-0414)
Supplement: Translation

# Translation: Percepção do discente de enfermagem: uso do *Escape Room* no ensino de habilidades de liderança

**DOI:** 10.1590/0034-7167-2023-0414pt

**Authors:** Chennyfer Dobbins Abi Rached, Emili Amani Alves da Cruz, Maria Helena Cardoso da Mota, Caroline da Silva Fonseca Paulo, Giovanna Micucci Pires Amaral, Daniela Lika Nakajima

**Affiliations:** I Universidade de São Paulo. São Paulo, São Paulo, Brasil

**Keywords:** Liderança, Ensino, Gamificação, Enfermagem, Percepção

## Abstract

**Objetivos:**

compreender a percepção do discente de enfermagem quanto ao uso do *Escape Room* para o desenvolvimento de habilidades de liderança.

**Métodos:**

estudo qualitativo exploratório-descritivo, com 97 graduandos de enfermagem. Foram aplicadas sessões de jogos de *Escape Room*, totalizando dez. Posteriormente, foi realizado um *debriefing* e análise feita pelo método do Discurso do Sujeito Coletivo, por meio da técnica do grupo focal. Os procedimentos éticos foram respeitados.

**Resultados:**

as percepções dos discentes sobre o uso dessa metodologia para desenvolver habilidades de liderança foram positivas e significativas. Destacam-se a eficácia do jogo no ensino e o desenvolvimento dessas habilidades na prática da enfermagem.

**Considerações Finais:**

o jogo foi uma metodologia ativa efetiva na abordagem do conteúdo proposto, promovendo satisfação e fácil assimilação.

## INTRODUÇÃO

Nos dias de hoje, o sistema de saúde vem evoluindo para um novo formato baseado na atenção primária focada na assistência domiciliar e na comunidade, além dos serviços nos hospitais. Essa mudança fortalece a atuação do enfermeiro como líder da equipe de enfermagem^(1)^. Entretanto, poucos profissionais da enfermagem defendem que a liderança é requerida para desenvolver uma melhor qualidade no serviço e uma prestação de cuidados eficientes. Já a literatura da educação em enfermagem sugere uma necessidade de desenvolver a consciência dos profissionais da área sobre a necessidade da liderança, assim como a ampliação dos métodos pedagógicos de desenvolvimento de habilidades de liderança nos enfermeiros^(2)^.

Em 2018, ocorreram mudanças nas Diretrizes Curriculares Nacionais (DCN) dos cursos de enfermagem, direcionando a formação do profissional para o cumprimento dos princípios e diretrizes do Sistema Único de Saúde (SUS). Para que isso ocorra, o processo de ensino-aprendizagem necessita de uma mudança, com foco em estimular o potencial criativo, a autonomia e o autogerenciamento da aprendizagem. Assim, recomenda-se a utilização de uma conduta inovadora que proporciona habilidades de inserção na realidade do trabalho em saúde. Dessa forma, faz-se necessária a utilização de tecnologias diversificadas no processo de construção do saber^(3)^.

O *Escape Room* ou *breakout* é considerado um jogo sério, a partir da inserção dos jogadores em um espaço lúdico físico ou digital, com a finalidade de resolver um problema através de pistas. Por ser versátil, tem-se aplicado ao ensino para a aquisição de saberes e habilidades nas diversas áreas do conhecimento^(4)^. No Brasil, tais termos estão inseridos nesta metodologia emergente chamada gamificação, que tem como objetivo implementar elementos de jogos em atividades que não sejam os jogos, por exemplo, na sala de aula^(5)^. Assim, o *Escape Room* surge como uma ferramenta de ensino no processo de ensino-aprendizagem com experiências documentadas no ensino de diversas áreas, tais como saúde, ciências da computação^(6)^, matemática e linguagens^(7)^, entre outras, com resultados similares, sendo que os participantes se mostraram mais motivados e interessados nas atividades adaptadas aos determinados cenários.

Adentrando a realidade profissional dos enfermeiros, a estratégia do *Escape Room,* recentemente, foi escolhida para a realização de uma atividade de sensibilização e educação sobre prevenção de lesões por pressão em um hospital filantrópico de São Paulo, tendo a participação de diversos profissionais de saúde, como enfermeiros, técnicos em enfermagem, nutricionistas e fisioterapeutas^(8)^. Analisando os resultados e discussões produzidos pela pesquisa, foi possível visualizar como até na prática profissional essa estratégia possui uma efetividade importante e cumpre de maneira satisfatória com os objetivos que essa se propõe.

Além disso, estudos afora também demonstram a ampla usabilidade do *Escape Room*, utilizando-o para outras estratégias. Em uma universidade de engenharia da Itália, observou-se que, devido à dinâmica da universidade, os estudantes não socializavam muito entre si, fazendo com que não desenvolvessem aspectos cruciais para suas vidas pessoais e profissionais. Dessa maneira, a metodologia ativa foi aplicada visando justamente promover uma socialização entre esses, a fim de estimular trabalho entre equipe, capacidades de comunicação, entre outros. Ainda que seja proveniente de contextos diferentes, esse meio educacional também foi avaliado como muito satisfatório pelos participantes^(9)^.

Estudos apontam que há maior interesse em querer aprender mais sobre determinado assunto quando se utiliza um método lúdico^(4,9-10)^. Nesse aspecto, acredita-se que o jogo possa ser uma estratégia de aprendizado das habilidades de liderança durante a formação de enfermagem, posto que, de acordo com as DCN, a liderança é uma das competências essenciais para a formação do profissional de saúde que devem ser construídas ao longo de seu ensino superior^(11)^. Dessa forma, uma vez que os alunos estão ativamente encontrando formas de resoluções para um problema em comum dentro de um jogo *Escape Room*, eles também estão desenvolvendo habilidades e competências essenciais para sua atuação profissional.

Sendo assim, este estudo pretende responder á seguinte questão de pesquisa: qual a percepção do discente de enfermagem quanto ao uso do *Escape Room* para o desenvolvimento de habilidades de liderança?

## OBJETIVOS

Compreender a percepção do discente de enfermagem quanto ao uso do *Escape Room* para o desenvolvimento de habilidades de liderança.

## MÉTODOS

### Aspectos éticos

Todos procedimentos éticos foram respeitados, para o Brasil, com base na Resolução n^o^. 466/2012. O projeto foi aprovado pelo Comitê de Ética da Escola de Enfermagem da Universidade de São Paulo (EEUSP). Foi apresentado aos participantes, explicitando a importância da colaboração para o aprofundamento e a compreensão sobre tema e esclarecendo a liberdade e a voluntariedade de participação sem qualquer tipo de prejuízo ou sanção. Todos os participantes assinaram o Termo de Consentimento Livre e Esclarecido (TCLE).

### Referencial teórico-metodológico

Foi utilizada a análise de Discurso do Sujeito Coletivo (DSC)^(12)^.

### Tipo de estudo

Este estudo qualitativo, com caráter exploratório-descritivo^(13)^, possibilitou aprofundar o conhecimento sobre o tema estudado e a exposição das percepções e opiniões dos sujeitos da pesquisa sobre a realidade vivenciada. Para a elaboração do manuscrito, foram seguidas as recomendações do *COnsolidated criteria for REporting Qualitative research* (COREQ)^(13)^, instrumento disponibilizado pela rede EQUATOR.

### Procedimentos metodológicos

Os dados foram coletados na sessão do *debriefing* pelo método grupo focal, que envolve uma abordagem de pesquisa^(14)^ que emprega recursos de estímulo, frequentemente atividades interativas, para promover e manter conversas em grupo. Essas conversas facilitam a troca de conhecimentos e experiências entre os participantes. Nesse sentido, foi realizado o jogo *Escape Room* e, ao término, foi realizada uma sessão de *debriefing* para compreender a percepção dos estudantes sobre aspectos que envolviam o trabalho em equipe e liderança desenvolvidos ao longo do jogo. Todas as atividades foram realizadas conforme o cronograma.

### Cenário do estudo

Teve como cenário, inicialmente, duas salas, ambas de reunião, com cerca de 20 m^2^, com capacidade para até dez pessoas, com portas de acesso à entrada e saída dos participantes e uma porta entre as duas salas, com mesa, cadeiras e alguns armários. As janelas das duas salas davam acesso a um corredor, por essa razão elas foram cobertas para manter a privacidade do jogo, dos participantes e dos pesquisadores. Depois, devido à maior facilidade, agilidade e experiência dos jogos, a simulação passou a ocorrer em uma sala de simulação de uma casa de 20 m^2^ com capacidade para até dez pessoas. A sala contava com: portas de acesso à entrada e saída dos participantes; três cômodos distintos (sala de estar, banheiro e quarto) com portas de acesso para cada um; sala de estar contendo sofá, mesa de jantar, pia, geladeira, máquina de lavar, tanque e gabinetes; banheiro contendo chuveiro, vaso sanitário e pia; quarto contendo cama, escrivaninha e um armário; janelas que davam para a parte de fora da sala. Havia uma sala de controle e observação integrada à sala de simulação, na qual, por meio de dois *notebooks*, transmitiam as imagens e os sons por intermédio do *Google Meet^®^*, presentes nos dois cenários, igualmente. Portanto, foi preciso preparar as salas para que elas recebessem o jogo. Dessa maneira, as pesquisadoras se reuniam cerca de 30 minutos antes para fazer a preparação da sala. As janelas foram cobertas com papel kraft, para que não houvesse interferência do meio externo. O estudo ocorreu na escola de enfermagem de uma Instituição de Ensino Superior, de caráter público, situado no centro da cidade de São Paulo, SP, Brasil.

### Fonte de dados

O estudo foi desenvolvido com 97 alunos, selecionados de forma intencional, que vivenciavam a graduação de enfermagem. O quantitativo de participantes foi distribuído entre sete e dez participantes em cada jogo, totalizando dez jogos. Logo após a finalização do jogo, esse mesmo grupo de participantes era direcionado para um grupo focal, no qual se discutiam as percepções acerca do *Escape Room*. O total de participantes foi delimitado pela caracterização da repetição das falas, possibilitando atingir a compreensão do fenômeno estudado. Os participantes foram identificados por número sequencial da fala no grupo focal (*suj_1, suj_2*). Os critérios de inclusão compreenderam alunos da graduação em enfermagem, estudantes da escola de enfermagem entre o primeiro e o último semestre da graduação, de forma que não havia uma homogeneidade dos grupos focais, já que os alunos dos diferentes semestres eram colocados em grupos aleatoriamente. Mesmo com a não homogeneidade da amostra, foram aplicados os mesmos desafios e critérios de avaliação do jogo para todos os estudantes que participaram da pesquisa. A única variável entre os grupos era a quantidade de pistas a mais que cada grupo recebia, de acordo com o tempo que estavam levando para concluir o *Escape Room*. Os critérios de exclusão abrangeram quaisquer pessoas que não estivessem na graduação de enfermagem da escola de enfermagem.

Os participantes eram recrutados por meio de convites pelos meios digitais (*WhatsApp^®^* e e-mail) e interpessoais nas salas de aula. Para a participação no jogo, era necessário que a pessoa manifestasse o interesse, para que os preceptores organizassem o calendário de jogos de acordo com a demanda e disponibilidade dos participantes. Se houvesse desistência no dia do jogo, eram realocados para algum outro dia que haveria o jogo novamente. Para selecionarmos nossa fonte de dados, optamos por captá-los por meio de um *debriefing*, aplicando-se a técnica do grupo focal e de um questionário, pois, ao mesmo tempo que o primeiro explorou a subjetividade dos resultados da atividade e os instigou a refletir, o outro serviu de auxílio para analisarmos essa subjetividade de uma maneira mais objetiva, captando tópicos que buscávamos discutir e para nos permitir ter uma visão estatística melhor.

### Apresentação do jogo

As sessões foram organizadas de forma que havia um tempo hábil para a realização tanto do *Escape Room* quanto do momento do *debriefing* para aplicação da técnica do grupo focal. Todos os preceptores jogaram o *Escape Room* em um espaço específico, compreendendo a dinâmica e forma de avaliação.

Foi estabelecido um contato prévio antes do início do jogo com os participantes para uma breve explicação do jogo, sua duração e forma de monitoramento dos preceptores, assinatura do TCLE e para comunicar que, ao final do jogo, seria realizada a sessão de *debriefing*. O jogo tinha um tempo máximo de 60 minutos para ser finalizado, composto por duas etapas, cada qual com sua história e pistas, que foram distribuídas na sala. Conforme o jogo acontecia, os pesquisadores sempre estavam atentos às dificuldades e habilidades que os grupos apresentavam, de tal maneira que, para alguns grupos, foram dadas mais pistas (soltas por debaixo da porta) para a completude do jogo. Os desafios e as pistas do jogo estavam relacionados com a história fictícia de um professor de laboratório que deixou com que um vírus contagioso se espalhasse em seu laboratório, que agora está trancado com os alunos dentro, e eles precisam descobrir as pistas deixadas pelo professor para sair de dentro dessa sala e se salvar da contaminação. Ao final do jogo, todos os participantes desse mesmo jogo se reuniam com os pesquisadores para que o momento do *debriefing* fosse realizado.

### Coleta e organização dos dados

Nesta pesquisa, contou-se com a realização de dez sessões grupais, com agendamento acordado entre os participantes no período de dezembro de 2022 a março de 2023, realizadas logo após o acontecimento do *Escape Room*. Não foram coletados dados sociodemográficos. Somente os pesquisadores realizaram a coleta de dados, havendo previamente um treinamento e discussão entre os mesmos para serem definidos os melhores métodos para essa finalidade. As respostas verbais dos participantes não foram gravadas, somente anotadas de maneira mais objetiva, tendo em vista que também foi utilizado um questionário escrito que foi preenchido por todos os participantes do grupo focal. O planejamento do grupo focal foi feito de acordo com as seguintes etapas: composição (participantes, equipe de coordenação, recrutamento); ferramentas (ambientação e guia de temas); operacionalização (estruturação); e avaliação.

### Etapas do trabalho

#### Composição

Participantes: 97 estudantes do curso de graduação em enfermagem que participaram de uma sessão completa do *Escape Room*. Alguns dos participantes tinham relacionamento prévio com a equipe, no entanto isso não influenciou na aplicação do grupo focal;

Equipe de coordenação: composta por uma docente da escola de enfermagem que tem o foco de pesquisa em liderança, mais quatro discentes de enfermagem que participaram de um treinamento prévio para a coleta de dados. A equipe era responsável por direcionar a discussão acerca do jogo com os participantes e anotar tais percepções;

Recrutamento: o convite para o grupo focal se deu antes do início do *Escape Room*. Não foi dado critério de livre escolha para a participação do grupo focal e nem o direito de recusa ou desistência. Foram explicados aos participantes os objetivos, os métodos, os riscos e os benefícios do grupo focal, assim como a temática que seria discutida.

#### Ferramentas

Ambientação: o grupo focal ocorreu na mesma sala da primeira parte do jogo no primeiro cenário. Depois, quando houve a mudança de cenário, o grupo focal aconteceu na sala de controle. Os participantes e os moderadores ficaram dispostos no grupo focal em uma mesa redonda, todos sentados. O acolhimento dos participantes se deu logo ao final do jogo, e os pesquisadores presentes acolhiam os participantes na sala para a realização do grupo focal;

Guia de temas: os temas do guia foram elaborados pelos autores a partir da análise dos principais temas abordados quanto ao assunto “liderança”. Também foram analisados os principais fatores que foram necessários para uma boa conclusão da dinâmica, após sessão teste com os moderadores e autores. As perguntas norteadoras foram: como você percebe o trabalho em equipe (saíram sozinhos ou individualmente, como foi o trabalho colaborativo)? Houve conflitos? Como foram resolvidos? Existiu um líder na equipe e como foi sentir essa liderança? Qual a percepção em relação aos membros equipe? Tiveram um papel ativo na solução dos problemas que seria conseguir a meta do jogo que era a fuga?

#### Operacionalização

Estruturação: houve dez encontros para o grupo focal com os mesmos participantes que compuseram o grupo para o *Escape Room*. Cada encontro durou em torno de 30 minutos. Como o grupo focal ocorreu logo após os jogos, não houve a aplicação de alguma dinâmica “quebra-gelo” entre os participantes. Os pesquisadores ficaram responsáveis pela moderação do grupo, solicitando aos discentes a recordar e detalhar o conteúdo do jogo, trazendo percepções e habilidades aplicadas. Depois que os participantes esgotaram as memórias, foram discutidos aspectos e conceitos de liderança colaborativa. As falas dos participantes eram transcritas pelos pesquisadores concomitantemente ao grupo focal. Na sala onde ocorreu o grupo focal, só havia a presença dos participantes e dos pesquisadores responsáveis pela moderação.

#### Avaliação

Foi utilizado o guia de avaliação aplicado ao fim de cada sessão com o intuito de identificar como foi a condução do grupo focal na visão dos participantes. Esse instrumento foi composto de questões relacionadas ao local e horário, operacionalização (objetivos, tema do jogo, condução da discussão participativa e síntese), papel do moderador e dos observadores. As possibilidades de resposta eram em uma escala Likert, sendo ela: 0 - Não se aplicou de maneira alguma; 1 - Aplicou-se em algum grau ou por pouco tempo; 2 - Aplicou-se em um grau considerável ou por uma boa parte do tempo; 3 - Aplicou-se muito ou na maioria do tempo. Foram ministrados uma aula e um treinamento para aplicação do instrumento. A análise dos resultados apontou a adequação do método.

#### Construção do instrumento

O instrumento foi elaborado pelos próprios autores, e o conteúdo do instrumento foi submetido à validação por especialistas de liderança (juízes). A construção e a validação foram baseada em três polos (teórico, empírico (experimental) e analítico (estatístico)). O polo teórico fundamentou as propriedades e dimensões dos objetos de estudo. A avaliação foi realizada de acordo com três critérios (clareza, relevância e aparência). Os juízes atribuíram valores de 1 (nada relevante) a 5 (muito relevante) para cada um dos itens. O valor 1 foi a pior nota, e o valor 5, a melhor. Já aqueles que tinham valores maiores ou iguais 4 indicam que o item correspondeu ao objetivo proposto. Para a análise de conteúdo do instrumento, foi utilizado o Índice de Validade de Conteúdo (IVC), que mede a proporção ou porcentagem de juízes que estão em concordância sobre determinados aspectos do instrumento e de seus itens. O escore do índice foi calculado por meio da soma de concordância dos itens que foram marcados por “4” ou “5” pelos especialistas. O nível de concordância considerado aceitável foi um IVC para cada item maior ou igual a 80%. O IVC^(15)^ foi usado para determinar a validade dos itens do questionário. Os escores de IVC variaram de 0,74 até 1,00, e o total do IVC foi de 0,93, indicando a adequada validade do conteúdo. O pré-teste realizado no *Escape Room* foi constituído por 12 participantes.

#### Análise de dados

Os dados foram coletados e transcritos imediatamente após as sessões de *Escape Room*, durante a realização de uma mesa redonda com os voluntários. Um dos moderadores que acompanhou o jogo foi responsável pela transcrição, que foi posteriormente compartilhada com os autores. A identificação e a seleção dos temas ocorreram a partir da análise das falas dos voluntários. Essas informações foram analisadas e interpretadas seguindo o método DSC^(12)^, que permitiu reconstruir, por meio de recortes de distintos discursos individuais, uma espécie de quebra-cabeça, representando os discursos-síntese considerados essenciais para expressar o pensamento coletivo dos participantes deste estudo sobre o fenômeno investigado.

## RESULTADOS

De acordo com as falas durante os grupos focais, os estudantes perceberam que o *Escape Room* possibilita a aplicação de habilidades de liderança, como trabalho em equipe, comunicação, tomada de decisão, planejamento, criatividade e persistência. Observou-se que esse método pode ser utilizado como uma intervenção educacional no ensino para as habilidades de liderança em enfermagem, visto que as reflexões trouxeram aspectos que favorecem o exercício da liderança das equipes de enfermagem, como engajamento, trabalho em equipe, gestão de tempo e de conflitos. Na fase de interpretação, os dados referentes às habilidades de liderança que são aplicadas durante o *Escape Room*, sob a perspectiva dos estudantes que participaram do jogo, foram agrupados por categorias de Ideias Centrais (ICs). As mesmas serão apresentadas a seguir na forma de DSC.

### Trabalho em equipe

Nesta IC, os entrevistados demonstraram perceber que o trabalho em equipe é essencial para alcançar o objetivo do jogo. Grande parte dos participantes foi capaz de valorizar a relação com o outro, o trabalho colaborativo, e compreender os aspectos da gestão de problemas, o impacto nas nossas ações, bem como de que maneira pode afetar o âmbito do trabalho, provocando, assim, reflexões quanto às mudanças no comportamento tanto na forma de liderar quanto na forma de operacionalizar os processos de trabalho.

*Quando nos ajudamos e pensamos em grupo, desvendar a pista foi mais rápido.* (suj_11)

*Pensando em grupo é bem fácil e ágil do que nós pensássemos sozinhos.* (suj_84)

*Quando todo mundo começou a trabalhar junto, foi quando a coisa fluiu.* (suj_90)

### Liderança

No tópico de liderança, parte significativa dos participantes relatou, inicialmente, não ter notado o destaque de um indivíduo como líder, mas sim que em vários momentos diferentes pessoas se destacaram guiando o grupo para a solução dos enigmas propostos. Muitos relataram também que sentiam falta da abordagem sobre liderança durante a graduação e que gostaram de ter um contato prático com o tema.

*Achei muito bom trazer uma nova metodologia para o ensino da enfermagem. A liderança é um tema vago para nós alunos, e pensar que o líder precisa alcançar metas e inspirar seguidores é algo que tem todo sentido no Scape.* (suj_41)

*Na hora do jogo, acabei não percebendo muito por causa da empolgação, mas agora pensando ter um líder foi fundamental para que o trabalho em equipe desse certo.* (suj_12)

*Quando se começa a entender o papel do enfermeiro durante a graduação, a liderança surge até como uma habilidade necessária, mas é bem difícil aprender isso na teoria. Com o Escape, parece que fez mais sentido.* (suj_19)

### Sentimentos em relação à estratégia

Estudantes verbalizaram o sentido positivo e agregativo da estratégia de gamificação para a formação universitária no que tange à competência da liderança.

*Parece que, quando tava todo mundo engajado nessa atividade, começaram a aparecer várias estratégias pra gente resolver o problema, foi bem interessante.* (suj_25)

*Vai para além do líder, eu penso. Acho que a estratégia surgiu com todos unidos trabalhando juntos pensando em uma maneira de chegar a uma resolução.* (suj_76)

### Comunicação

Em todas as sessões, houve participantes que evidenciaram uma boa comunicação, expondo satisfação quanto a como o grupo conversava quando se depararam com um obstáculo.

*A liderança precisa de comunicação e relacionamento interpessoal. Senti que é fundamental para o alcance do objetivo do jogo, acho que tem relação com a prática da liderança.* (suj_87)

*No comecinho do jogo, todo mundo estava um pouco calado, cada um fazendo alguma coisa, mas a gente percebeu que só ia conseguir desvendar as pistas se a gente se comunica, então, pra mim, acho que aquela pessoa que teve a iniciativa de iniciar uma comunicação efetiva com todo mundo pode se tornar um bom líder.* (suj_56)

### Tomada de decisão

Os resultados mostraram que os participantes perceberam a importância do líder no processo de tomada de decisão sob pressão, destacando a necessidade de decisões rápidas para alcançar os objetivos. Além disso, foi observado que as decisões tomadas foram colaborativas, com todos os membros do grupo contribuindo com sugestões e opiniões.

*As decisões foram todas em conjunto. Alguém dava uma sugestão e todos davam sua opinião.* (suj_15)

*No momento em que todo mundo tava se ajudando, tomar as decisões ficou mais fácil, já que o líder do nosso grupo manifestou o interesse na opinião de cada pessoa para dar o próximo passo.* (suj_66)

### Planejamento

Durante a experiência, os alunos de enfermagem relataram duas perspectivas diferentes em relação ao planejamento. Alguns participantes mencionaram que o processo foi caótico, indicando que não houve um planejamento efetivo durante o jogo. Por outro lado, outros alunos notaram que uma pessoa assumiu o papel de líder e se encarregou do planejamento, o que resultou em uma experiência mais organizada e focada.

*Nossa, não tivemos planejamento, um caos.* (suj_22)

*Senti que uma pessoa assumiu o planejamento e prendeu a atenção, favorecendo o planejamento de onde iríamos.* (suj_66)

### Criatividade

Os discentes enfatizaram a importância da criatividade para desvendar pistas e superar desafios no jogo. A colaboração em grupo também foi destacada como essencial para impulsionar a criatividade, já que cada membro trouxe ideias únicas, enriquecendo a resolução dos problemas.

*Precisamos ser muito criativos para desvendar as pistas, pensar em grupo ajudou também, porque cada um tinha uma ideia.* (suj_74)

*Todo mundo ajudou todo mundo, foi uma chuva de ideias uma hora.* (suj_69)

### Persistência

Os participantes relataram situações que evidenciaram sua persistência diante dos desafios apresentados. Um dos participantes expressou ter enfrentado momentos de confusão e desmotivação, chegando a considerar desistir. No entanto, no momento em que a equipe conseguiu desvendar uma pista, essa sensação de vitória proporcionou “um momento mágico e gratificante”. Outro discente mencionou que também enfrentou dificuldades significativas, ao ponto de sentir vontade de desistir e deitar na cama. Entretanto, sua colega de equipe desempenhou um papel importante ao incentivá-lo e motivá-lo a continuar. A sensação de conquista e vitória ao final do jogo evidenciou a importância da cooperação e do apoio mútuo entre os membros da equipe.

*Viajei no jogo, não estava entendendo nada, aí queria desistir, mas, quando o alarme do codificador tocou que acertamos, foi um momento mágico de vitória.* (suj_24)

*Teve uma hora que eu queria deitar na cama, meu, muito difícil, mas minha colega me puxou e disse “vamos”, acho que tem a ver com o papel de líder.* (suj_69)

## DISCUSSÃO

Aplicando o *Escape Room*, percebe-se primordialmente como o trabalho em equipe é um ponto forte do jogo e percebido pelos jogadores. Na atividade, juntos, eles descobrem pistas, solucionam quebra-cabeças e realizam tarefas em uma ou mais salas, a fim de alcançar um objetivo específico (que geralmente é escapar da sala) em uma quantidade determinada de tempo^(16)^. Sua prestabilidade fica evidente ao se comparar essa metodologia ativa do *Escape Room* com as mais tradicionais que envolvam recursos humanos em grupos. Os participantes se mostraram ativamente envolvidos nas atividades e o nível de energia foi significativamente maior^(17)^.

Mais interessante ainda é associar o trabalho em equipe à profissão, em que diversos enfermeiros hoje em dia se deparam com conflitos com a equipe, desconhecimento sobre quem são seus colegas de trabalho e suas capacidades, insegurança de se relacionar, tudo isso muitas vezes associado como produto das problemáticas anteriormente citadas, pois um ambiente de trabalho sobrecarregado e muitas vezes extremamente rotativo traz muitas dificuldades para a dinâmica grupal. Dessa maneira, o *Escape Room* mostrou que tem capacidade de fazer as pessoas se conhecerem melhor entre si, as capacidades de cada uma e se unirem para um mesmo objetivo, demonstrando ampla usabilidade para os graduandos de enfermagem^(18)^.

Além disso, mais do que ser capaz de trabalhar em equipe, o enfermeiro muitas vezes encontra-se na posição de liderá-la. O conceito de liderança se dá pelo processo de interações, em que os líderes são responsáveis por influenciar aqueles que os seguem, de forma a executar ações com o objetivo de atingir objetivos em seus próprios contextos^(19)^. No jogo aplicado, foi possível observar que muitos participantes experienciaram seu autoconhecimento quanto à liderança e também à sua capacidade de identificá-la em outras pessoas.

Dessa maneira, desenvolver desde a graduação suas capacidades de liderança é um pilar fundamental para que muitos cenários sejam evitáveis. O *Escape Room* inclui todos os estudantes como potenciais líderes, e isso é importante, pensando em um gestor e sua equipe, uma vez que torna as relações mais horizontais. Um bom líder fornece oportunidades para seu grupo e reconhece seus esforços^(20)^, formador de opiniões nas equipes, gerenciando pessoas, e o comportamento e o poder praticados influenciam diferentes aspectos do ambiente de trabalho e das relações. Desenvolver a liderança dos estudantes de graduação auxilia na sua atuação como profissional. Liderança é abordada durante os cursos de enfermagem em algumas disciplinas de maneira superficial, por isso propiciar essa oportunidade aos estudantes foi uma estratégia para a formação de profissionais mais preparados e conscientes para a criação de locais de trabalho positivos que promovam o bem-estar e o engajamento dos trabalhadores. Esse jogo colocou todos em posições ativas para busca de conhecimento e raciocínio crítico, na tentativa de fazer com que as pessoas se sintam reconhecidas e confiantes, tornando seu engajamento melhor^(20)^.

Consequentemente a isso, há diversas habilidades que o *Escape Room* busca aprimorar nos jogadores, e uma delas é a criatividade, que favorece a comunicação eficaz, visão ampliada para resolução de problemas, estimulando o raciocínio crítico. Nas sessões dos jogos, foi possível analisar, nas percepções dos estudantes, a presença dessas habilidades e sua satisfação com a estratégia educacional quanto a isso, demonstrando sua efetividade.

A criatividade é uma das habilidades de liderança essenciais na enfermagem. O enfermeiro criativo identifica e busca soluções inovadoras, melhorando os cuidados de saúde e motivando a equipe tanto de enfermagem quanto de saúde para oferecer uma assistência segura e de qualidade. Os líderes de enfermagem podem usar a criatividade para melhorar os processos operacionais/assistenciais, elaborando protocolos eficientes, introduzindo novas tecnologias e implementando estratégias para reduzir iatrogenias^(21-24)^. Por isso, a comunicação eficaz é fundamental no contexto da saúde, uma vez que auxilia na garantia de que as informações sejam transmitidas de maneira clara e compreensível para a equipe^(25)^.

O enfermeiro toma decisões tanto em relação à gestão do cuidado quanto em relação à gestão da equipe, resolvendo problemas complexos e buscando criar um ambiente de trabalho que promova a segurança do paciente e o bem-estar da equipe. Nesse aspecto, a criatividade e a liderança se entrelaçam na busca de resolver os desafios do cotidiano da enfermagem, como alocação de recursos, otimização de processos de assistência ao paciente, gerenciamento de conflitos, entre outros que fazem com que a criatividade seja essencial para identificar soluções inovadoras para os problemas^(24-27)^.

Além disso, o processo de decisão tem grande relevância sob a efetividade e o sucesso dentro das instituições, pois ele é essencial dentre as habilidades do líder, sendo um processo com potencial solucionador de problemas, permitindo o raciocínio sobre qual caminho traçar e quais escolhas trarão maior benefício para a equipe e organização^(28-29)^. No *Escape Room*, por diversas vezes, os estudantes são colocados em posições em que a tomada de decisão é necessária para resolução do jogo, por isso há um estímulo importante quanto a isso, notado por muitos deles em nossa experiência.

O pensar crítico sobre o cuidado, com embasamentos teóricos e práticos, cabe à equipe de enfermagem, para ser capacitada com inovação e criatividade sobre melhores métodos, meios e, principalmente, para a preparação acerca de tomada de decisões para este, o que implica diretamente a qualidade assistencial no atendimento a quem os serviços são prestados. Tudo isso está inerentemente ligado à capacidade do planejamento, uma função privativa do enfermeiro, de acordo com as divisões sociotécnicas de trabalho e que, como habilidade, é um pilar fundamental para que os processos dentro do serviço de saúde possam ocorrer com êxito e organização^(30)^.

Assim, nota-se como tal fato traz à tona quantas são as perspectivas e faces de situações muito relacionadas à habilidade em si de realizar o planejamento em enfermagem, além de como isso afeta situações terceiras também, como a prevenção de erros, o uso correto e eficiente de recursos, a satisfação de quem recebe o cuidado^(31)^. É interessante que muitos estudantes não perceberam, de antemão, todo seu planejamento de estratégias de jogo, em que foram utilizadas várias das habilidades anteriormente citadas, fazendo com que, a partir do *debriefing*, pudessem refletir e analisar como suas capacidades se conectam de maneira importante para realizar trabalho efetivo.

Por fim, a utilização do *Escape Room* demonstrou-se peça fundamental para o crescimento pessoal e coletivo dos estudantes. O *Escape Room* educativo segue os princípios da gamificação, isto é, ele é fundamentado no funcionamento, na estética e no pensamento lúdico para entreter as pessoas, fomentar as atividades, proporcionar o aprendizado e a resolução de problemas com dinâmicas e mecanismos particulares dos jogos^(7)^.

Em suma, a gamificação mostra ter um importante potencial para o aumento da motivação dos estudantes, corroborando para uma melhor compreensão e resolução de problemas^(32)^ que encontrarão em seus caminhos. Isso ocorre ao utilizar as habilidades que serão capazes de desenvolver com a estratégia e, assim, colaborar para a formação de profissionais mais capacitados e sistemas de saúde mais efetivos.

### Limitações do estudo

O estudo foi limitado pela origem dos estudantes utilizados, de forma que todos vieram da mesma instituição e apenas do curso de enfermagem, o que pode ter influência nos resultados relacionados à metodologia da gamificação.

### Contribuições para a área de enfermagem

A pesquisa trouxe evidências da potencialidade das metodologias de ensino com modelagens comportamentais para o desenvolvimento da liderança colaborativa em enfermagem, identificando possibilidades para a aplicabilidade dessa metodologia em disciplinas do curso de graduação, não apenas de enfermagem, mas de outros cursos e outras temáticas, como o trabalho interprofissional, cultura organizacional, entre outras, fortalecendo a formação com qualidade.

## CONSIDERAÇÕES FINAIS

Através do *Escape Room*, foi observada uma facilitação no aprendizado da liderança. Os estudantes de enfermagem perceberam a liderança como competência gerencial essencial para o desenvolvimento do trabalho em equipe, resolução de problemas complexos e conflitos. As maiores dificuldades de aprendizado estavam relacionadas às competências de persistência e planejamento, entretanto, no geral, os estudantes demonstraram satisfação em relação à metodologia proposta e uma fácil assimilação dos conteúdos sugeridos.
